# 

**Published:** 1902-09

**Authors:** 


*THE BRISTOL MEDICO-CHIRURGICAL SOCIETY.*

### CONTENTS.

| | PAGE |
|---|---|
| **The Reputation of the Hotwells (Bristol) as a Health-Resort.** By L. M. GRIFFITHS, M.R.C.S. Eng. ... ... ... | 193 |
| **A Case of Ascites due to Thrombosis of the Hepatic Veins.** By THEODORE FISHER, M.D., M.R.C.P. ... ... ... | 209 |
| **Paralysis of the Accommodation—an a posteriori view of Diphtheria.** By W. M. BEAUMONT, Esq., M.R.C.S., &c. | 213 |
| **Stereoscopic X-ray Representation, with an example.** By WILLIAM COTTON, M.A., M.D., D.P.H. ... ... | 218 |
| **The Treatment of Certain Deformities, more especially of the Nose, by the Subcutaneous Injection of Paraffin.** By J. PAUL BUSH, C.M.G., M.R.C.S. | 220 |
| **Notes on Three Pathological Specimens.** By C. E. S. FLEMMING, M.R.C.S., L.R.C.P. ... ... ... ... ... ... ... | 224 |
| **The Unreliability of the Microscope in the Diagnosis of Malignant Disease.** By C. HAMILTON WHITEFORD, M.R.C.S., L.R.C.P. ... ... ... ... | 226 |
| **On the Use of Morphia in Uræmia.** By T. M. CARTER, M.R.C.S., L.R.C.P., and F. H. EDGEWORTH, M.B., B.Sc., B.A.... | 231 |
| **Progress of the Medical Sciences:** | |
| **Medicine.** R. SHINGLETON SMITH, M.D., B.Sc. Lond. ... ... ... ... ... ... | 235 |
| **Surgery.** T. CARWARDINE, M.S. Lond., F.R.C.S. Eng. ... ... ... ... ... ... | 240 |
| **Dermatology.** HENRY WALDO, M.D., C.M. Aberd. ... ... ... ... ... ... | 243 |
| **Reviews of Books** ... ... ... ... ... ... | 248 |
| **Editorial Notes** ... ... ... ... ... ... | 270 |
| **Notes on New Drugs and Preparations for the Sick** ... ... ... ... ... ... | 275 |
| **The Library of the Bristol Medico-Chirurgical Society** ... .. ... ... | 277 |
| **Meetings of Societies** ... ... ... ... ... | 282 |
| **Local Medical Notes** ... ... ... ... ... | 286 |

